# Varicella Zoster Reactivation Manifesting as Serpiginous Peripheral Keratitis and Disciform Keratitis Occurring After Necrotizing Fasciitis in an Immunocompromised Male: A Case Report

**DOI:** 10.7759/cureus.40787

**Published:** 2023-06-22

**Authors:** Vinita Gupta, Himani Pal, Sucharita Das, Divya Sindhuja Pathuri, Madhubari Vathulya

**Affiliations:** 1 Ophthalmology, All India Institute of Medical Sciences, Rishikesh, IND; 2 Ophthalmology, Government Doon Medical College, Dehradun, IND; 3 Ophthalmology, Oculoplasty, Orbit & Ocular Oncology, Institute of Medical Sciences and Sum Hospital, Bhubaneswar, IND; 4 Ophthalmology, All India Institute of Medical Sciences, Mangalagiri, Manglagiri, IND; 5 Plastic and Reconstructive Surgery, All India Institute of Medical Sciences, Rishikesh, IND

**Keywords:** peripheral serpiginous ulceration, rheumatoid arthritis, peripheral ulcerative keratitis, disciform keratitis, necrotizing fasciitis, herpes zoster ophthalmicus

## Abstract

Reactivation of herpes zoster ophthalmicus (HZO) can present as corneal involvement without any precedent neuralgia or characteristic herpetic rash. This form of HZO can be the first manifestation of reactivation of varicella zoster virus and can masquerade as peripheral ulcerative keratitis. A 45-year-old male treated for necrotizing fasciitis (NF) one month back presented with painful diminution of vision in the right eye (RE) for two weeks without any associated vesicular rash or neuralgia. On examination, best-corrected visual acuity in RE was 2/60 with non-marginal upper lid defect, and multiple linear contracture scars involving the upper lid, right temple, and preauricular region. There were associated peripheral corneal ring infiltrates, disc-shaped central stromal edema, and reduced corneal sensation. The patient had a history of chicken pox in childhood and was recently diagnosed with seropositive rheumatoid arthritis (RA). Though corneal scrapings were negative on Tzanck smear, a presumptive clinical diagnosis of herpetic disciform keratitis was made, and the patient was started on oral and topical acyclovir with steroids. This was confirmed with improving clinical course and detection of herpes zoster DNA on polymerase chain reaction from corneal scrapings. Lid reconstruction for associated lid defect was performed using paramedian forehead flap, which was remodeled at 16 weeks. Our case, a seropositive RA patient, had reactivation of varicella zoster manifesting as peripheral serpiginous and disciform keratitis activated after NF. There are a few case reports of periorbital NF following HZO in immunocompromised patients. However, till date, no case of HZO occurring after periorbital NF has been reported. Also, in our case, reactivation of HZO presented as disciform and serpiginous keratitis without any precedent herpetic rash or neuralgia.

## Introduction

Herpes zoster is a prevalent viral disease that inflicts substantial morbidity and associated healthcare and socioeconomic burdens [1]. It represents reactivation of varicella zoster virus in the host and shows variable clinical presentation and complications, which are potentially life-threatening. Herpes zoster ophthalmicus (HZO) is a reactivation of the varicella zoster virus involving the ophthalmic division of the fifth cranial nerve. Herpes zoster affects 20% to 30% of the population at some point in their lifetime, and approximately 10% to 20% of these individuals will have HZO [2]. Common risk factors for herpes zoster are age > 50 years, immunosuppression, infections, mental stress, rheumatic disorders, and use of disease-modifying drugs. Stress and psychological factors have also been shown to heighten the risk of herpes zoster [2,3]. It has been found that trauma also increases the risk of zoster at the trauma site [4]. Ocular involvement occurs in approximately 50% of herpes zoster patients in the absence of prompt antiviral therapy, causing significant morbidity in both normal and immunocompromised patients [2]. Kahloun et al. found adnexal involvement in 58.8%, anterior uveitis in 60.7%, keratitis with corneal hypoesthesia in 31.4%, oculomotor nerve palsy in 5.8%, and optic neuritis with optic disc edema in 1.9% as ocular manifestations of HZO [3]. Reported corneal complications of HZO include punctate epithelial keratitis in 51%, early pseudodendrites in 51%, anterior stromal infiltrates in 41 %, sclerokeratitis in 1%, kerato-uveitis/endotheliitis in 34%, serpiginous ulceration in 7%, delayed corneal mucous plaques in 13%, disciform keratitis in 10%, neurotrophic keratitis in 25%, and exposure keratitis in 11% [5]. Treatment for HZO includes prompt initiation of antiviral agents for all patients, as well as supportive care for symptom management [6]. Other adjunct therapies, such as antibiotics, topical or systemic corticosteroids, and corneal epithelial debridement, are considered on a case-by-case basis.

Necrotizing fasciitis (NF) is a rare infection that spreads rapidly along the subcutaneous soft tissue planes. The annual incidence of NF ranges from 0.4 to 15.5 cases per 100,000 in various regions of the world [7-11]. It usually involves the extremities, abdominal wall, and groin, and only 10% involve the head and neck. Involvement of eyelids is even rarer [12,13]. Risk factors for developing periorbital NF include alcoholism (26%), diabetes mellitus (10%), rheumatologic disease (7%), systemic malignancy (1%), and systemic corticosteroid use (1%) [14]. Other risk factors include upper respiratory illness, acute or chronic bacterial sinusitis, recent ocular or periocular infection, and systemic infections. Necrosis in periorbital NF becomes quickly visible following infection since eyelid skin is thin, with little subcutaneous fat [13,14]. There are a few case reports of periorbital NF following HZO in immunocompromised patients [15,16]. We describe here a case of HZO, which was re-activated after periorbital NF in an immunocompromised seropositive rheumatoid arthritis patient and manifested as peripheral serpiginous ulceration and disciform keratitis without any precedent herpetic rash or neuralgia.

## Case presentation

**Figure 1:**
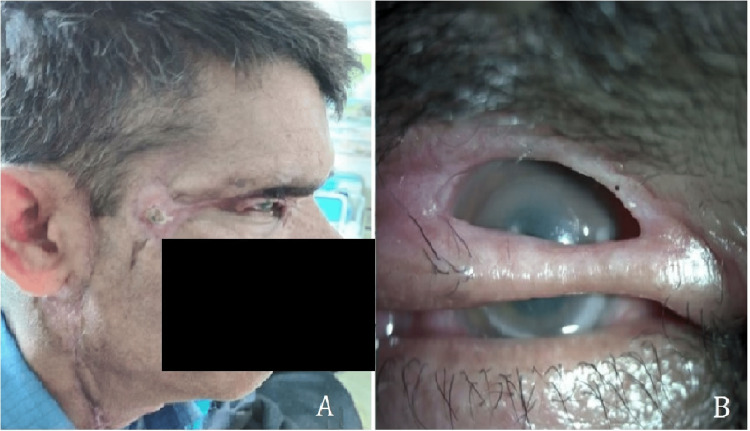
External photographs of the face and eyelids showing (A) multiple linear contracture scars involving the right upper lid, temple, preauricular region, and neck, and (B) non-marginal upper lid defect with incomplete closure of eyelid and corneal exposure.

**Figure 2:**
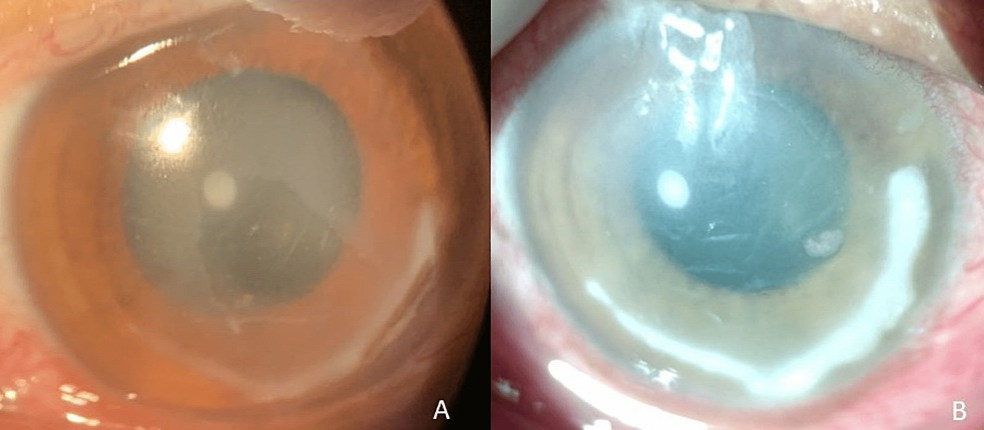
Slit lamp biomicroscopic corneal photographs of the right eye. (A) Diffuse illumination showing peripheral corneal ring infiltrate extending from 3’o to 7’o clock position with disc-shaped central stromal edema with central epithelial defect at time of presentation. (B) Diffuse illumination showing increasing central stromal edema with Descemet’s membrane folds and peripheral ring infiltrate over the next 48 hours.

**Figure 3:**
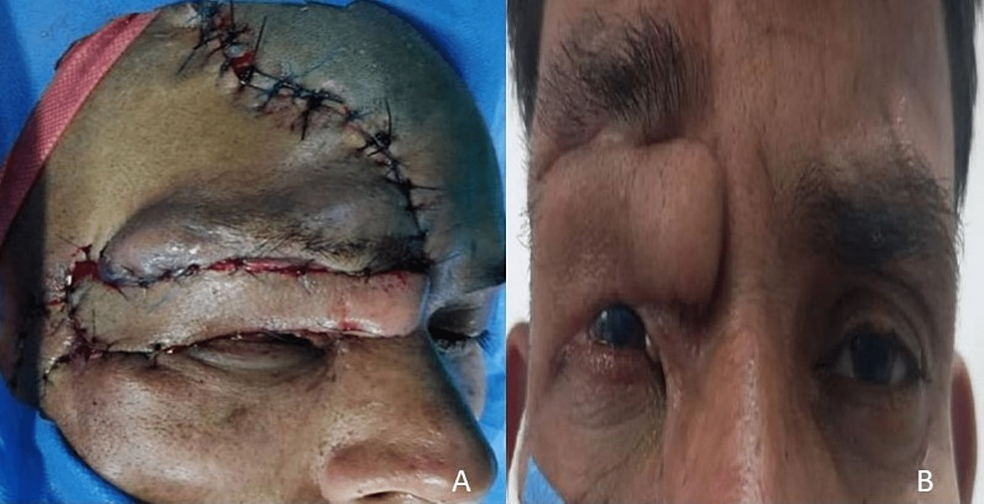
External photographs of the face and eyelids showing right paramedian upper lid flap reconstruction. (A) Intra-operative appearance at the end of surgery. (B) Post-operative appearance after four weeks.

**Figure 4:**
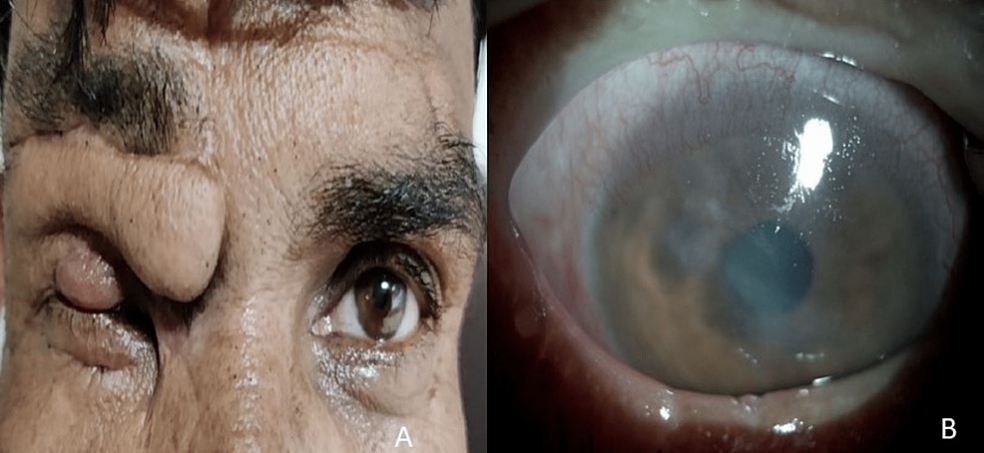
At 16 weeks. (A) External photograph of the face and eyelids showing remodeled paramedian upper lid flap. (B) Slit lamp biomicroscopic corneal photograph in diffuse illumination of the right eye showing healed corneal scar.

A 45-year-old male farmer presented with complaints of painful diminution of vision in the right eye (RE) for two weeks with associated redness and photophobia in the RE. There was no preceding history of any vesicular rash or any neuralgic pain in the surrounding area. The patient was a diagnosed case of deep vein thrombosis on treatment and had a prior history of treated pulmonary tuberculosis and chickenpox in childhood. He was apparently well and asymptomatic till two months back, when he had a fall from stairs after a bout of drinking with resultant minor skin abrasions and scratches on the right side of the head and neck with involvement of the right eyelid. At one-week post-fall, the patient presented with pain, discoloration, and swelling of the surrounding areas, accompanied by serosanguinous discharge. A diagnosis of periorbital NF was made at a different medical facility, and the patient underwent surgical debridement. Treatment included intravenous linezolid 600 mg administered every 12 hours, along with supportive therapy consisting of oral non-steroidal anti-inflammatory drugs, high-dose multivitamins, and vitamin C supplementation. There was no precedent vesiculomacular rash, no dermatomal distribution radicular pain, or any ocular complaints at that time. Microbiological analysis of the biopsied tissue had shown *Staphylococcus aureus* as the only causative organism, and histopathology had features suggestive of NF. On his presentation four weeks after the debridement for NF, ocular examination revealed best-corrected visual acuity (BCVA) of 2/60 in RE and 6/6 in the left eye (LE). There was a non-marginal upper lid defect with multiple linear contracture scars involving the right upper lid, temple, and preauricular region (Figures 1A, 1B). Slit lamp examination revealed a crescent-shaped infiltrate in the right nasal peripheral cornea extending from 3’o clock to 7’o clock position associated with thinning. There was accompanying disc-shaped central stromal edema with few keratic precipitates and reduced corneal sensation (Figure 2A). LE also showed superficial punctate keratopathy in inferior periphery with normal corneal sensation. Intra-ocular pressure by non-contact tonometry was 15 and 13 mm of Hg in RE and LE, respectively. Fundus was unremarkable in both eyes. With the clinical picture of peripheral ulcerative keratitis (PUK) with disciform keratitis, corneal scrapings were sent for routine microbiological analysis, and the patient was started on topical fortified tobramycin six hourly, a cycloplegic agent three times a day, and carboxymethylcellulose 0.5% two hourly along with application of therapeutic bandage contact lens for corneal protection due to a large contracted upper lid defect. The patient was also investigated for any underlying autoimmune disease. While awaiting results of laboratory analysis, the patient had worsening of clinical picture over 48 hours (increasing stromal edema, Descemet’s membrane folds, and enlarging epithelial defect (Figure 2B). At this point, with a provisional diagnosis of herpetic serpiginous ulceration and disciform keratitis, corneal scrapings from peripheral ring infiltrate were sent for Tzanck smear and polymerase chain reaction (PCR) analysis, and oral acyclovir 800 mg five times a day, eye ointment acyclovir 3% five times a day, and topical prednisolone 1% eight hourly were added. Autoimmune workup of the patient led to a diagnosis of rheumatoid arthritis with positivity for rheumatoid factor and antinuclear antibody (ANA), and the patient was also started on oral prednisolone 60 mg, methotrexate 15 mg once a week, and folic acid supplementation. Our diagnosis of herpetic keratitis was confirmed by the healing epithelial defect, decreasing stromal edema and ring infiltrate, and by PCR confirmation of herpes zoster virus (from corneal scrapings) over the next 72 hours. Right upper lid paramedian flap reconstruction was also performed in the next 48 hours for the non-marginal eyelid defect and contracture scars involving the eyelid for closure of eyelid defect (Figure 3A). Review after four weeks showed total healing of epithelial defect and no signs of stromal infiltrate with reconstructed upper lid (Figure 3B). At 16 weeks, remodeling of the reconstructed lid flap was performed for gaining upper lid excursion (Figure 4A). The patient at this point of time had BCVA of 6/36 with a healed corneal scar (Figure 4B). At the two-year follow-up, the patient is maintaining a good lid closure and a BCVA of 6/36 on ongoing immunosuppressants for rheumatoid arthritis.

## Discussion

NF and other necrotizing soft tissue infections have been reported as sequelae of primary varicella zoster infection (chicken pox), first described by Hutchinson in 1882 [17]. They can occur in children and less commonly in adults, but risk increases with age and immunosuppression. NF is divided into the following types based on microbiological culture [18]: Type I NF (70-80% cases) is synergistic or polymicrobial caused by mixed aerobic and anaerobic organisms, often bowel flora-derived, and is seen in immunocompromised patients. Type II NF (20-30% cases) is usually caused by single organisms, group A ß-haemolytic streptococci or staphylococci, which are skin- or throat-derived, and is seen in non-immunodeficient patients. Type III NF is commoner in Asia, caused by Gram-negative, often marine-related, organisms and *Vibrio* spp. mainly. Type IV NF is fungal, usually associated with trauma, and seen in immunocompetent patients.

Periorbital NF is uncommon because of the high vascularity of this area. Most common trigger for periorbital NF is penetrating or surgical trauma, and the risk of developing NF is high in patients with comorbid conditions. Patients with periorbital NF present with acute periorbital swelling associated with severe pain, erythema, and necrotic skin with fluid-filled bullae, which can further show extensive subcutaneous involvement. In addition, pattern of infections associated with periorbital NF is different from rest of the body. In a review of all cases of periorbital NF over 20 years, Amrith et al. found that type I NF is quite rare in the periorbital region [19]. Pure cultures were usually present in around 70% cases, more than what is found in craniofacial NF. Polymicrobial infections of type 1 NF were more common in cases where the predisposing factor is trauma including surgical trauma.

There are several case reports of systemic NF following herpes zoster infection [20,21]. Also, at the stage of bullae eruption, it may be confused as herpes zoster. Wright et al. reported a case of subacute NF of the posterior neck, which disguised as herpes zoster infection [22]. Cases of periorbital NF following HZO have also been described. Fung et al. described periorbital NF in a 63-year-old female with discoid lupus, who developed NF following cutaneous herpes zoster infection, which responded well to surgical debridement and appropriate antimicrobial therapy [15]. Cozzupoli. et al reported periocular NF following shingles in a 70-year-old female with Waldenstrom’s macroglobulinemia, who also had a history of keratoplasty for herpetic keratitis in the past. She underwent debridement and fasciotomy for NF but died eventually due to a mix of complications of the medical treatment for sepsis and comorbidities [16]. Saldana et al. also described a case of a 59-year-old male with HZO who developed periorbital cellulitis three days later, which spread down his face onto his chest, which was managed with debridement, intravenous acyclovir, and antibiotics [23].

However, all these have described occurrence of NF following herpes zoster. Till date only one case of a 74-year-old diabetic male has been described by Ha and Tyring, in which herpes zoster reactivation was preceded by NF [24]. Their patient had painful vesicles with unilateral radicular pain extending across the left side of chest and back in the region of the T5 dermatome, which had begun to emerge 7 days after resolution of NF. The authors highlighted that in their case, trauma induced by the wound and subsequent NF may have provided an adequate degree of stress to provoke the development of HZ and its associated post-herpetic neuralgia. Our case had reactivation of HZO, and this was preceded by periorbital NF. In our patient, who was already immunocompromised, trauma and subsequent development of NF, which required surgical debridement, acted as triggers for the reactivation of herpes zoster in the trigeminal ganglion. However, there was no rash on the forehead, eyelid, and tip of the nose, any atrophic hypopigmented scars (signs of previous zoster rash), or the characteristic neuralgic pain in our patient. Our case thereby highlights that HZO may be reactivated after trauma and NF and may manifest as serpiginous peripheral and disciform keratitis without any neuralgia or rash especially in immunocompromised patients.

Also, our patient presented with PUK (serpiginous ulceration) and disciform keratitis as the manifesting signs of reactivation of herpes zoster. Herpetic keratitis can masquerade as PUK especially in patients with immune system dysregulation causing diagnostic dilemma, as reported by Paridou et al. [25] in a case of a 47-year-old female with a history of arthritis and positive ANA. Our patient also had immune dysregulation and was diagnosed with seropositive rheumatoid arthritis with positive ANA.

## Conclusions

The concept of the latency and reactivation of the varicella zoster virus from the sensory nerve ganglion is well established. Ocular complications from HZO may occur in the absence of a skin rash (zoster sine herpete), and an astute clinician must recognize that some corneal manifestations may be due to zoster, even if the clinical setting does not offer the complete background. A search of the history, a careful clinical examination, and serologic tests may help in some of these cases, as in our case of an immunocompromised male who manifested with serpiginous peripheral keratitis and disciform keratitis as signs of varicella zoster reactivation occurring after periorbital NF.
